# The Role of Aquaporins in Plant Growth under Conditions of Oxygen Deficiency

**DOI:** 10.3390/ijms231710159

**Published:** 2022-09-05

**Authors:** Guzel Kudoyarova, Dmitriy Veselov, Vladislav Yemelyanov, Maria Shishova

**Affiliations:** 1 Ufa Institute of Biology of Ufa Federal Research Centre of the Russian Academy of Sciences, Pr. Octyabrya 69, 450054 Ufa, Russia; 2 Department of Genetics and Biotechnology, Faculty of Biology, Saint Petersburg State University, Universitetskaya em., 7–9, 199034 St. Petersburg, Russia; 3 Department of Plant Physiology and Biochemistry, Faculty of Biology, Saint Petersburg State University, Universitetskaya em., 7–9, 199034 St. Petersburg, Russia

**Keywords:** aquaporins, oxygen deficiency, coleoptile, elongation growth

## Abstract

Plants frequently experience hypoxia due to flooding caused by intensive rainfall or irrigation, when they are partially or completely submerged under a layer of water. In the latter case, some resistant plants implement a hypoxia avoidance strategy by accelerating shoot elongation, which allows lifting their leaves above the water surface. This strategy is achieved due to increased water uptake by shoot cells through water channels (aquaporins, AQPs). It remains a puzzle how an increased flow of water through aquaporins into the cells of submerged shoots can be achieved, while it is well known that hypoxia inhibits the activity of aquaporins. In this review, we summarize the literature data on the mechanisms that are likely to compensate for the decline in aquaporin activity under hypoxic conditions, providing increased water entry into cells and accelerated shoot elongation. These mechanisms include changes in the expression of genes encoding aquaporins, as well as processes that occur at the post-transcriptional level. We also discuss the involvement of hormones, whose concentration changes in submerged plants, in the control of aquaporin activity.

## 1. Introduction

Oxygen is necessary for the normal metabolism of plants, and its deficiency negatively affects almost all biochemical and physiological processes, ultimately leading to inhibition of plants growth and a decrease in agricultural crop yield. There are several native scenarios that are triggered by oxygen deficiency of different intensity (hypoxia and anoxia). Among main reasons are the formation of an ice crust, asphalt pavement, or, more often, flooding of plants as a result of heavy rain or excessive irrigation. Due to the poor solubility of gases in water (including oxygen), waterlogging of the rhizosphere leads to local root hypoxia, while complete submergence of a plant more often causes a much more severe effect such as total anoxia [1]. Under normal conditions, the availability of oxygen in the soil is ensured by the diffusion through well-developed air-filled pores and channels. Compaction and water saturation of soils are suggested to be the main barriers to soil oxygen transport [2]. In the case of local soil flooding when only the roots are under direct oxygen deprivation, O_2_ can come from the shoot through aerenchyma, whose enhanced development is one of the characteristic adaptive reactions of plants to flooding [3,4,5]. It is more difficult to survive submergence when the entire plant (root and shoot) is under water. In rare cases (transparent H_2_O), light can pass through a water layer, enabling photosynthesis and, thus, oxygen production by the leaves of fully submerged plants. This rarely occurs in natural conditions, since the water is most often opaque due to the presence of a large amount of suspended solids and dissolved colored material reducing water clarity [6]. The lack of oxygen can be also aggravated by the pathogenic action of soil biota [1].

As a result, plants experience disturbances in respiration and photosynthesis, and intoxication develops due to the accumulation of toxic metabolic products, particularly acetaldehyde and ethanol [7,8]. A lack of oxygen stimulates glycolysis, as well as lactic and alcoholic fermentation [8,9,10]. At the same time, the rapid activation of lactate dehydrogenase, observed in many plants, may be the cause of the initial acidification of the cytosol, and then, with time, alcoholic fermentation begins to predominate [10,11].

Under such conditions, plants adapt to hypoxia through two alternative strategies. The first relies on the decrease in the growth and metabolic rate, with a switch to anaerobic metabolism (low-oxygen quiescence syndrome, LOQS) [10,12]. Energy generated due to glycolysis and fermentation is mostly used for the synthesis of stress proteins involved in chaperone activity, membrane transport, and antioxidant defense [9]. The second alternative pathway for plants to tolerate their shoot submergence, on the contrary, depends on intensification of the shoot growth that serves to elevate the photosynthetic tissues above the water surface [6,12,13]. This strategy (low-oxygen escape syndrome, LOES) is implemented by many varieties of rice (deep-water), as well as the majority of wild hydrophytes adapted to grow in wetlands (e.g., *Callitriche platycarpa*, *Hydrocharis morsus-ranae*, *Nymphoides peltata*, *Ranunculus repens*, *R*. *sceleratus, Rumex crispus*, and *R. palustris*), as reviewed in [14,15]. The study of the mechanisms involved in plant growth activation under hypoxic conditions is important not only to find ways to improve plant resistance to oxygen deficiency, but also for a better understanding plant growth regulation in general. In this review, emphasis is placed on an aspect of this problem, which, in our opinion, has received insufficient attention. It consists in elucidating the role of water channels, aquaporins (AQPs), in the activation of plant shoot growth under conditions of oxygen deficiency.

## 2. Aquaporin Structure and Plant Growth

Plants, unlike animals, have the ability to rapidly and significantly increase their cell volume due to considerable water uptake. The length of the cells in this case can increase manifold. The dependence of the rate of cell elongation on the uptake of water is described by the equation R = L(ΔΨ), where R is the cell growth rate, L is the hydraulic conductance, and ΔΨ is the difference in water potential between the cell and external medium, which is in turn broken up into its two components, osmotic pressure and hydrostatic pressure [16]. Numerous investigations of cell elongation growth regulation focused on the mechanisms maintaining the level of osmotically active substances against the background of their dilution resulting from water uptake [17,18], and (perhaps even to a greater extent) attention was paid to the state of the cell wall, which provides its extension under the influence of the hydrostatic component of the water potential, i.e., turgor [19,20,21]. It is well proven that growth is a complex process in which all cell compartments are involved. Elongation is always accompanied by vacuolization. This highlights the importance of water flux not only through plasma membrane but also through the tonoplast. Since vacuoles occupy up to 90% of the plant cell volume, water is transported constantly through the tonoplast, and the intensity of the osmotic flow and the volume of vacuoles depend on the permeability of the vacuolar membrane [22,23].

Water can penetrate cell membranes both directly through the phospholipid bilayer [24] and indirectly through specialized water channels, aquaporins [25,26]. The concept of the ability of small molecules, such as water, to freely diffuse through the membrane is based on the ability of liposomes (artificial vesicles containing only lipids) to absorb water [27]. However, experiments with the expression of *Arabidopsis* aquaporin genes in a heterologous system (e.g., in *Xenopus* oocytes) showed an increase in permeability of cell membrane for water due to aquaporins [25]. It was revealed that, with an increase in the expression of genes encoding them, aquaporins are able to accelerate cell lysis in a hypotonic medium detected by changes in the optical density of the suspension [28,29]. This approach is widely used to demonstrate the ability of certain aquaporins to conduct water [30,31]. Modulation of AQP abundance through transcriptional approaches affects the hydraulic conductivity of plant biological membranes [32,33]. In comparison with mammals having 15 types of AQPs described so far (displaying 18 paralogs), a single plant species can represent more than 120 isoforms, providing the transport of different types of solutes [34]. In plants, water transporters have been found in all tested organs including roots, leaves, stems, flowers, fruits, and seeds [35]. AQPs in higher plants are classified into five subfamilies: plasma membrane intrinsic proteins (PIPs), tonoplast intrinsic proteins (TIPs), nodulin-26 like intrinsic proteins (NIPs), small basic intrinsic proteins (SIPs), and X intrinsic proteins/uncharacterized-intrinsic proteins (XIPs). AQPs are known as low-molecular-weight proteins of about 23–31 kDa [36] containing six transmembrane helices. Four AQP monomers assemble to form homo- or heterotetrameric complexes [31,34], with each monomer acting as an independent water channel. Features of the aquaporin tertiary structure enable the incorporation of these proteins into the hydrophobic layer of membranes due to the exposure of hydrophobic domains on the surface of the protein molecule of AQPs. At the same time, the presence in the molecule of a channel with a small radius lined with hydrophilic domains ensures the movement of small hydrophilic molecules along it (not only water, but also glycerol, ammonia, urea, lactic acid, hydrogen peroxide, different ions such as boron, silicon, and arsenate, and even some gases such as CO_2_ and O_2_ [35]). Intensive investigation of the structures of AQPs proteins and the genes encoding them revealed that AQPs are conserved transport proteins identified in each kingdom [37].

There is extensive evidence proving the importance of AQPs in growth and development in plants and even determining health and disease in the case of animals. A study of the localization of aquaporins in animals and plants revealed a high level of their presence in sites characterized by a high level of water flow (e.g., in the kidneys of animals [38] or in the cells around plant vascular bundles [39]. It has been noted that AQPs are not only important at a cellular level but also involved in the whole-plant functional water transport between shoot and root. Tobacco plants with antisense transformation with *NtAQP1* expression provided evidence for NtAQP1 function in cellular and whole-plant water relations. A decrease in cellular water permeability due to antisense construct positively correlated with transpiration rate, stem and leaf water potential changes, and decrement in growth intensity under extreme soil water depletion [40]. On the contrary, the increased levels of AQPs and the expression of aquaporin-encoding genes in roots correlated with higher water flow and elevated hydraulic conductivity of roots, e.g., during the photoperiod [41] or when air heating increases transpiration rate [42]. Changes in PIP transcript amounts in the morning were synchronous with the changes in plant hydraulic conductance [43].

## 3. The Growth under Normoxic Conditions and the Role of Water Transporting Aquaporins

A constant water supply is important for growing tissues required for maintenance of turgor pressure upon cell enlargement [44]. Water entering the cells during growth passes through the plasma membrane and tonoplast (vacuolar membrane). Plasma membrane (PIP) and tonoplast (TIP) aquaporins are involved in this process [45]. PIP aquaporins are divided into two groups: PIP2 and PIP1. Expression of class II aquaporin genes in a heterologous system increased membrane permeability for water, while PIP1 aquaporins showed weak ability to conduct water. Nevertheless, it was demonstrated that PIP1;1 has high water channel activity when co-expressed with PIP2, and that PIP1–PIP2 random heterotetramerization not only allows PIP1;1 to arrive at the plasma membrane, but also results in an enhanced activity of PIP2;1 [31,46]. These results suggested the importance of both PIP1 and PIP2 aquaporins for the control of water movement across the plasma membrane.

TIP aquaporins have a special function in the process of cell elongation growth, since they ensure the absorption of water by vacuoles, providing an increase in cell volume. TIPs basically act as regulators of the intracellular water flow, thereby defining cell turgor [47]. Therefore, they are given special attention when debating the role of aquaporins in the process of cell growth [44,48]. TIP aquaporins play an important role in seed germination [49]. Their abundance is positively correlated with the formation of leaf vacuoles [50] and is typical for rapidly growing cells, especially in roots [51]. Multiple TIP isoforms with water channel activity have been shown to co-express abundantly in root cells, but it remains to be shown which (if any) of these TIP isoforms carries a growth-specific function [52].

The admitted discrepancy of results focusing on plant growth ability (see below) can be explained by the presence of a large number of representatives of the family of both PIP and TIP aquaporins in different plant species, which differ in their localization in tissues of shoots and roots. For example, the *Arabidopsis* genome contains 38 sequences with homology to aquaporin [53], while 33 aquaporin genes were identified in rice genome [54], and a comprehensive search revealed that the barley MIP family comprises at least 40 aquaporins [55]. The diversity of aquaporins may be the reason for the difference in the results of their detection in growing cells when only some of the genes and proteins of this family were analyzed. Elucidation of the role of aquaporins in the regulation of cell growth is important in connection with the debate about the involvement of membrane water permeability in the regulation of cell elongation [43]. Since ΔΨ (the difference between the water potentials of the cell and its environment) depends on the hydraulic conductivity of cell membranes, it is considered as an indicator of membrane permeability to water. High ΔΨ values indicate that hydraulic conductivity is low and water uptake limits cell growth, while low ΔΨ values indicate high hydraulic conductivity and that growth is primarily limited by cell-wall parameters [56]. Measurements carried out on individual cells showed low ΔΨ values, indicating that their membranes are highly permeable for water (cell Lp), and that hydraulic conductivity does not limit cell elongation [16]. However, a study on elongating barley leaf cells revealed the existence of a significant (>0.1 MPa) ΔΨ, being indicative of some hydraulic (co-)limitation of cell expansion, in growing cells [57]. The model proposed by Calderia and co-authors suggests that a hydraulic process may account for the rapid changes in LER upon changes in evaporative demand or soil water content [43]. The hydraulic conductivity of cortical cells decreased in the elongating tissue and increased slightly during growth recovery in response to changes in availability of water and elongation rate [58]. The role of aquaporins increases during the transport of water from its source (xylem and phloem vessels) to individual growing cells, since water overcomes a large number of membranes along this path, and the hydraulic resistance of all crossed membranes is summed up [52]. The authors suggested that cell Lp and growth-dependent expression of AQPs in leaves match more the need for water transport through tissue than for expansion of an individual cell.

The original investigation, which employed growing maize suspension cultured cells, showed that the Pf (membrane osmotic water permeability coefficient) was significantly increased at the end of the logarithmic growth phase and during the steady-state phase compared to the lag phase. A positive correlation between AQP abundance in the plasma membrane and the cell Pf was elucidated at cellular level [59].

Manifold elevation of *ZmTIP* expression during the post-germination period was much more intensive in comparison with the increase in ZmPIP abundance [60]. The analysis of early maize seedling growth revealed that overdevelopment TIPs might be detected in the plasma membrane. Moreover, this effect corresponded to a higher intensity of water flow through cell membranes. Such a complicated profile of PIP and TIP expression, as well as its different redistribution between plasma membrane and tonoplast over development, demands additional investigation.

Roots are the most convenient and widely used objects for studying growth, since cell division and elongation occur in different zones of the root tip. Roots are dominant organs in AQPs expression [61]. PIPs and TIPs are enriched in the growing regions of the root tip, where more growing cells and tissues exist [50]. It was demonstrated with in situ hybridization and immunolocalization that VvPIP1 and VvPIP2 proteins and expression of their genes were localized evenly in the cortex and vascular tissues of the root tip, but showed lower signals in the cortex of mature root regions [62]. However, the relationship between aquaporin gene expression and growth cannot always be traced. It was shown that overexpression of PIP1 aquaporin of *Vicia faba* in *Arabidopsis* promoted the growth of primary and lateral roots (LRs) [63]. Overexpression of poplar *PtoPIP1;1* in *Arabidopsis* accelerated cell growth in both the leaf and root [64]. On the contrary, *Arabidopsis* antisense plants with decreased expression of *AtPIP1a* and *AtPIP1b* had more abundant roots compared to the control plants under normal condition [65].

Stem growth, including juvenile hypocotyls, is significantly based on a cell’s ability to elongate. This process was shown to be regulated by AQPs. The importance of PIPs and TIPs was shown for different plant species such as *Arabidopsis*, pea, maize, tulip, castor bean, and rice [50]. Moreover, ectopic expression of APQs genes from one plant species in another was followed by intensification of shoot growth. As an example, transgenic tobacco (*Nicotiana benthamiana*), expressing rice *OsPIP1;3*, was characterized not only by intensive shoot growth but higher photosynthesis rates, Lpr, and water-use efficiency [66]. A similar positive role was determined for vacuolar AQPs. Accordingly, the overexpression of *AtTIP5;1* in *Arabidopsis* resulted in hypocotyl cell elongation [67]. In adult plants, the ratio between root and shoot growth is an important parameter which was found to be under control of AQPs. This phenomenon was proven by 1.5-fold elevation of the shoot/root ratio in rice plants with overexpression of barley *HvPIP2;1* [68]. This coincides with the suggested aquaporin involvement in long-distance water transport (xylem function), as well as in short-distance transcellular water flow, and in intracellular osmotic adjustment.

The importance of leaf growth is based on a variety of biochemical and physiological processes such as photosynthesis, respiration, and transpiration. A number of investigations have demonstrated that genes encoding PIPs and TIPs are natively expressed more intensively in the most elongated zones of *Arabidopsis* and maize leaves [51,69]. Ectopic overexpression of *Vicia faba VfPIP1* and *Panax ginseng PgTIP1* in *Arabidopsis*, as well as citrus *CsTIP2;1* in tobacco, significantly intensified leaf growth and increased the size of leaf mesophyll cells in all cases [50]. Such an increase is known to be dependent on the AQP-induced elevation of the hydraulic conductivity of leaves (Lpl) characterizing leaf elongation zones [70].

**Figure 1:**
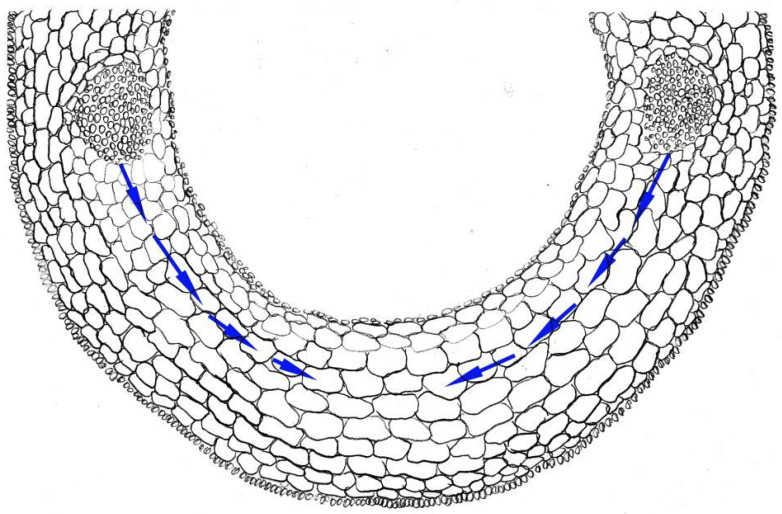
Scheme of cross-section of rice coleoptile. Arrows indicate the water pathway from vascular bundles to elongating cells. This figure was drawn by Dr. G. Sharipova on the basis of microscope observation.

Another classic model for studying elongation growth is cereal coleoptiles (Figure 1). These are juvenile organs that protect the leaf during germination. Inherently longer coleoptiles are supposed to be an advantage in many cases, such as the protection from high temperature and dense environment [71,72]. Currently, a large number of factors have been identified that regulate coleoptile elongation at the transcriptional and post-translational levels [73,74].

To clarify the molecular mechanisms of elongation growth in these cells, the theory of acidic growth was proposed back in the 1970s [75,76,77]. It implies the activation of the proton ATPase of the plasma membrane and subsequent acidification of the cell wall, leading to an increase in its plasticity. It coincides with an alteration in the membrane potential value, as well as the direction of transport of different ions through plasma membrane. Subsequently, this primary stage is accompanied and followed by the activation of a number of cell systems, which leads to intensive elongation growth [78,79]. Unfortunately, we failed to find clear evidence revealing the importance of AQP activity, especially in the determination of coleoptile growth.

Given examples strictly define the role of water-transporting AQPs (PIPs and TIPs), located in the plasma membrane and tonoplast, in the determination of water flow over elongation growth.

## 4. Shoot Growth under Hypoxia and the Role of Aquaporins

Aquaporins may be considered as factors limiting growth during hypoxia. The decrease in hydraulic conductivity is one of the first effects recorded during flooding [80]. This reaction leads to a decrease in stomatal conductance and transpiration. A decline in hydraulic conductivity allows explaining the paradoxical nature of stomatal closure in plants experiencing water excess during flooding, which is normally a characteristic response of plants to water deficiency. The decline in hydraulic conductivity is believed to be due to acidification of tissues brought about by the changes in metabolism resulting from hypoxia. Cytosolic acidification closes the AQP pore and reduces the membrane permeability to water [81] due to the protonation of a conserved histidine residue in aquaporin molecules following a drop in cytoplasmic pH [82]. Inhibitors of cytochrome pathway respiration can be used to mimic oxygen deprivation [83]. Although this effect has been registered in differentiated cells, it is obvious that hypoxia should reduce the hydraulic conductivity of growing cells in the same way. Consequently, the opinion that the membrane permeability of individual cells is too high and, thus, cannot limit the growth of cells by elongation [56] is obviously incorrect under hypoxic conditions. Hypoxia-induced decline in hydraulic conductance makes it a real limiting factor for elongation growth.

Meanwhile, shoot elongation is an important stage of plant survival known as the LOES strategy under submergence. Limited data have been obtained with a focus on coleoptile elongation growth under conditions of oxygen deficiency. In the case of flooding, the initial step of rice seedling development is intensification of coleoptile elongation [84]. Once coleoptiles reach the water surface, the formation of the aerenchyma occurs, which provides the developed seedling (root and primary leaf) with oxygen [85]. Some data indicated a typical quantitative characteristic of coleoptile elongation intensity [84,86]. Further genetic mapping analyses identified several quantitative trait loci (QTL) associated with anaerobic germination and early seedling growth under submergence [87,88]. Several QTLs (4–13 at different chromosomes) responsible for germination and further coleoptile growth under anoxia were found in a number of investigations [88]. Among candidate genes involved in anaerobic germination of tolerant rice were genes encoding anaerobic metabolism, cell-wall plasticity, hormone signaling, etc. [87,88,89]. Nevertheless, AQPs were not listed among them.

Furthermore, it is necessary to take into account that, during elongation of submerged shoot organs (e.g., coleoptiles), absorption of water by the cells directly from the solution is complicated due to the low water permeability of the layer of the cuticle covering coleoptiles or stem [90]. Therefore, water is flows to growing cells from the vascular bundles (there are only two of them in a coleoptile of rice), while crossing the membranes of a large number of cells, which leads to an increase in the contribution of aquaporins to the control of cell extension growth.

The hypoxia-induced decline in the activity of aquaporins raises the question of how acceleration of elongation growth, enabling the lifting of photosynthetic tissues above the water surface, can occur during whole-plant submergence. It can be assumed that the flow of water into growing cells during hypoxia may be facilitated by compensatory mechanisms that can maintain activity of aquaporins. Although an answer to this question requires additional experiments, in this review, we discuss the mechanisms that affect the ability of aquaporins to conduct water. To achieve this, we consider in more detail the factors influencing the activity of aquaporins.

## 5. Factors Affecting the Activity of Aquaporins

According to conventional wisdom, the activity of aquaporins as representatives of membrane transporters is generally controlled at the transcriptional and post-translational level. It is necessary to admit that quite a number of investigations have reported that the ability to modulate the expression of genes encoding aquaporins is the most powerful mechanism to control their activity. Transcription upregulation resulted in increased abundance of AQP proteins [33,50,91]. However, a direct correlation between the expression of aquaporin genes and abundance of proteins encoded by them could not always be traced [92]. Thus, under osmotic stress, the expression of a number of PIP aquaporins in the roots of maize plants increased tenfold, while the level of proteins encoded by them increased no more than 1.5 times [93]. It can be assumed that a high expression level of aquaporins compensates for the possible destruction of proteins induced by stress.

There are few and contradictory data on the effect of hypoxia on the expression of aquaporin genes, which can be explained by the fact that the differences in the strategies of plant adaptation to hypoxia were not always taken into account. A stable expression of aquaporins and hypoxia responsive genes in adventitious roots was linked to maintaining hydraulic conductance in tobacco (*Nicotiana tabacum*) exposed to root hypoxia [94]. In *Sorghum*, the transcript abundance of genes encoding several TIP and PIP aquaporins was differentially altered in response to waterlogging stress due to their tissue-specific roles [95]. AQPs were found to be downregulated in some studies [96]. However, higher transcript abundance of one PIP and two TIP aquaporins coupled with higher Lpr was detected in root tips of *Agave deserti*, in wet soil [97].

Even fewer studies have investigated the expression of aquaporin genes in elongating shoot cells during hypoxia. Aquaporins have been shown to be involved in rapid internode elongation of deep-water rice. Expression of two *OsTIP* and three *OsPIP* aquaporins was significantly enhanced by submergence, supporting the quick elongation of internodes [98]. However, it is still questionable which factors are involved in this up- or downregulation.

Coming to the post-translational level of regulation, one can notice that the employment of a number of model objects convincingly demonstrated that the permeability of water channels may be regulated primarily as a result of phosphorylation and dephosphorylation of serine and threonine residues of aquaporins [33,99,100]. These processes are controlled by certain protein kinases [101,102] and affect the tertiary structure of proteins and the size of the water channel (pore). Several sites for phosphorylation were determined in aquaporin protein. However, it was demonstrated that Ser256 phosphorylation was necessary for normal water transport across the plasma membrane, while phosphorylation of Ser264 and Ser269 did not show the same requirement [103]. Phosphorylation and dephosphorylation of plasma membrane aquaporins (and, thus, AQP activity) have been shown to be Ca^2+^- and pH-dependent [35,104]. In some investigations, AQPs were even suggested to have pH-sensing qualities [105]. Changes in cytosolic pH and Ca^2+^ concentration during oxygen shortage are well documented [106,107,108,109].

The presence of disulfide bridges between cysteine residues also plays an important role in maintaining the activity of aquaporins. One way to experimentally reduce the activity of aquaporins is to destroy disulfide bridges with heavy metals (mercury, silver) [110,111] or reactive oxygen species (ROS) [112]. The latter mechanism is considered to be more specific. This was demonstrated in experiments where an increase in the ROS level was modeled using Fenton’s reagent (a mixture of peroxide and divalent iron ions), which provides the formation of a hydroxyl radical as a result of the reaction of peroxide with iron ions [112]. A decrease in the activity of aquaporins induced by Fenton’s reagent led to reduction of hydraulic conductivity and a drop in water potential under air heating [42]. Oxygen deprivation, as well as reoxygenation, is known to stimulate ROS production [113].

Nonetheless, the effect of peroxide and other reactive oxygen species depends on their concentration: high concentrations of ROS inhibited aquaporins [114,115], while low concentrations of peroxides increased their activity [116]. Inactivation of ROS prevented increases in hydraulic conductivity and the level of aquaporins under the influence of abscisic acid (ABA) [117].

Another way to modulate the activity of aquaporins is their rapid traffic to the proper membranes [118]. This mechanism procures a rapid increase in the permeability of membranes for water without contribution to its synthesis or modification. The general mechanisms controlling AQPs traffic to the target membrane and their export out of the ER have not truly been discovered. A recent discovery elucidated that PIP2 primary protein sequences were found to carry the so-called diacidic motifs involved in the mechanisms of exit from the ER [119]. Another possible method of regulation is that the path of PIPs to the PM is under the control of SNAREs (soluble *N*-ethylmaleimide-sensitive factor attachment protein receptor, SYP121 isoform) of the syntaxin family [120,121]. This type of AQPs traffic was found to be very sensitive to stress factors, such as salinity, for both PIP and TIP representatives [118]. Moreover, intercellular traffic of PIP showed the ability to undergo lateral diffusion in the plasma membrane [122]. Such diffusion might correspond to the plasma membrane interaction with the cell wall and actin filaments [123,124]. Here, a complicated mechanism of endocytosis followed by AQPs degradation also has to be mentioned. The functioning of these complex multicomponent systems will inevitably be different according to tissue type, developmental stage, stress, etc.

Thus, a significant number of mechanisms might be involved in AQP regulation, and almost all of them are likely to occur under oxygen deficiency. Hypoxia and stronger anoxia induce cytosolic acidification, elevation of Ca^2+^ cytoplasmic concentration, alteration in ROS level, etc. [106,107,108,109,113]. However, available data are insufficient to conclude which of them is the most important.

## 6. Hypoxia, Hormones, Growth, and the Role of Aquaporins

Taking into account that growth is a process intensively regulated by plant hormones, the final part of our review is focused on the possible involvement of plant hormones in the regulation of AQP activity under oxygen deficiency. The role of hormones in the regulation of cell elongation during hypoxia has been discussed [10,15,125]. However, the involvement of hormones in the regulation of aquaporin activity in connection with the activation of shoot elongation during hypoxia has received little attention. Therefore, in discussing this problem, we involve data on the effects of hormones on the level of aquaporins in a broader aspect.

### 6.1. Effects of Ethylene under Hypoxia

The greatest amount of information about the role of hormones in the implementation of low-oxygen escape syndrome during hypoxia concerns ethylene [13]. Promotion of shoot extension by ethylene is mostly linked with cell-wall loosening provided by ethylene-dependent stimulation of enzymes involved in the process [15]. In the context of the problem we are discussing, it is important that, according to some data, ethylene is able to increase the expression of aquaporin genes [92,126], which can contribute to the activation of shoot growth. Ethylene significantly enhances root hydraulic conductivity by increasing plasma membrane permeability, permitting more water to cross the cells [127]. Increased water transport in hypoxic seedlings exposed to ethylene was explained in terms of enhanced aquaporin activity, probably due to a direct effect of ethylene on the phosphorylation of aquaporins [128]. The problem is that during hypoxia, ethylene accumulates not only in plants through implementing the strategy of avoiding oxygen deficiency by activating shoot elongation (LOES plants), but also in those using the opposite strategy of growth inhibition [129]. In addition, ethylene-sensitive factors (ERF) are involved in a recently discovered mechanism of oxygen sensing and response to its deficiency [130]. This mechanism is implemented via the Cys–Arg/N-end rule pathway through the oxygen-dependent degradation of a number of transcription factors, primarily group VII ethylene response factors (ERFVII). These transcription factors are recognized by the E3 ligase, which leads to their degradation with the participation of the 26S proteasome complex. A low oxygen concentration increases the stability of ERFVII, which leads to the launch of reactions that provide plant adaptation to flooding [131]. The problem is that this mechanism can upregulate the *HCR1* gene (hydraulic conductivity of root 1-Raf-like MAPKKK) that negatively controls hydraulic conductance, which is likely to be due to changes in AQPs activity. HCR1 accumulates and is functional under combined O_2_ limitation and potassium (K^+^) sufficiency [132]. The ambiguity of the effect of ethylene on hydraulic conductivity during flooding is explained by the fact that the response to oxygen deficiency depends on many concomitant factors, e.g., on the concentration of potassium or ethylene, as well as on the ability of ethylene to inactivate nitric oxide, which is also involved in the response of plants to hypoxia [133]. One way or another, the available information indicates the prospects for further study of the role of ethylene in the regulation of aquaporin activity under oxygen deficiency. The mechanism of action of ethylene during hypoxia may also be due to the interaction of ethylene with other hormones, whose concentration can be influenced by ethylene [134]. Thus, ethylene increases the concentration of gibberellins (GAs) [135], reduces ABA [13], and affects the distribution of auxins [136].

### 6.2. Gibberellins under Hypoxia

The role of gibberellins in the activation of stem elongation during hypoxia has been discussed in connection with the data on the accumulation of these hormones under flooding conditions, as well as their ability to accelerate shoot elongation due to the inactivation of DELLA (a known inhibitor of cell elongation growth [60]). Thus, GAs are credited with an important role in stimulating shoot elongation under hypoxia, but not at the expense of their possible effect on hydraulic conductivity or AQP activity. Although a review addressing functional aquaporin diversity in plants [137] gave many examples of the effects of GAs on aquaporins, there is little information about their role in the regulation of AQPs activity during flooding. Recently published work also showed that aquaporin AtTIP5;1 is an essential target of GAs promoting hypocotyl cell elongation in *Arabidopsis thaliana* under excess boron stress [138]. However, we failed to find information on the effect of GAs on aquaporins during hypoxia. At the same time, this does not mean that the role of GAs in the regulation of aquaporin activity during hypoxia is insignificant. Rather, this is evidence of insufficient knowledge of this issue, highlighting prospects for further research.

### 6.3. Abscisic Acid (ABA) and Hypoxia

By analogy with the role of ABA in the regulation of stomatal closure during drought [139], abscisic acid is supposed to be involved in the inhibition of transpiration during flooding. However, it was not possible to register an increase in the concentration of this hormone in the xylem during flooding, and its role in the regulation of stomatal behavior during flooding was not proven [140]. However, the involvement of ABA in the response of plants to flooding has been actively discussed [15,141]. In flooded LOES plants, a decrease in the concentration of ABA was detected [13], and the known ability of this hormone to inhibit growth, acting as an antagonist of GAs and maintaining the stability of DELLA, made it possible to associate a reduced concentration of ABA with growth activation [142]. The decrease in ABA concentration during hypoxia may be due to the action of ethylene, which can inactivate ABA via its conjugation [143] and downregulate its biosynthesis [15,125]. Nevertheless, in plants that are not characterized by stimulation of shoot elongation during hypoxia, a decrease in ABA concentration was not recorded [6], although (see above) ethylene accumulation occurred. Plants sensitive to oxygen deprivation accumulate a higher amount of more ABA during submergence or total anoxia [125,144].

In the context of the discussed problem of the regulation of aquaporin activity, it is of interest that there is more information about the effect of ABA on the activity of aquaporins than about any other hormone. An ABA-induced increase in both the level of aquaporins [145] and the expression of the genes encoding them, as well as their activation as a result of phosphorylation and traffic from the cytoplasm to the membranes, has been repeatedly shown [26]. However, during flooding, ABA is not likely to be involved in the activation of aquaporins during the acceleration of shoot elongation, since its concentration decreases in this case (see above). Nevertheless, uncertainties regarding this issue remain. Despite the decrease in the level of ABA, the level of its receptor increases during flooding [146].

### 6.4. Auxins and Flooding

Data on the effect of submergence on the content of auxins are contradictory. There is information on the submergence-induced decline in the level of indoleacetic acid (IAA) in petioles of *R. palustris* [147]. At the same time, anoxia-induced accumulation of IAA in wheat and rice seedlings has been shown, as well as the relationship of plant tolerance with oxygen deprivation [144,148,149]. The role of auxins in the activation of shoot elongation during flooding has been discussed mainly in connection with the ability of this hormone to influence the accumulation of osmotically active substances and the extensibility of the cell wall [150,151]. The possible effect of auxins on the activity of aquaporins is difficult to discuss, since there is very limited information on this subject. Most often, the same study is cited, where a decrease in the expression of aquaporins under the influence of auxins was demonstrated [152]. However, it was shown that treatment of plants with an auxin transport inhibitor prevented the increase in aquaporin expression registered during hypoxia in the absence of this inhibitor [153]. These results indicate that the normal distribution of auxins during hypoxia plays an important role in the induction of aquaporin expression.

## 7. Conclusions

A decrease in hydraulic conductivity, which is one of the characteristic consequences of hypoxia, indicates that this factor is limiting for cell growth by elongation during flooding. Since low-oxygen escape syndrome is based on the stimulation of shoot elongation during flooding, it is obvious that there must be a mechanism to compensate for the decline in hydraulic conductivity during hypoxia. The role of such a compensatory mechanism may consist of an increase of the ability of aquaporins to conduct water, which is indirectly evidenced by the little data on an increase in the expression of some aquaporin-coding genes during hypoxia and a change in the concentration of phytohormones under hypoxia, potentially capable of influencing the activity of aquaporins. At the same time, the amount of experimental data in this regard is extremely limited, which dictates the need for further research in this direction. Expanding our understanding of the regulation of aquaporin activity during hypoxia is a promising approach, not only for deepening knowledge about the ways to increase plant resistance to flooding, but also for a general understanding of the regulation of cell extension growth.

## Data Availability

Not applicable.
